# Overcoming Recurrent Isolated Sleep Paralysis: A Case Report of Integrative Management With Yoga, Meditation, and Vitamin D3 Supplementation

**DOI:** 10.7759/cureus.76626

**Published:** 2024-12-30

**Authors:** Dattaprasad Sawant, Neha Kamble

**Affiliations:** 1 Community Medicine, Ram Krishna Medical College Hospital and Research Centre, Bhopal, IND; 2 Community Medicine, Seth Gordhandas Sunderdas (GS) Medical College, King Edward Memorial (KEM) Hospital, Mumbai, IND; 3 Community Medicine, Terna Medical College, Navi Mumbai, IND; 4 Community Medicine, Grant Medical College and Sir JJ Group of Hospitals, Mumbai, IND

**Keywords:** holistic care for patients, sleep-paralysis, sleep-wake disorder, vitamin-d deficiency, yoga therapy

## Abstract

Recurrent isolated sleep paralysis (RISP) is a rare but distressing condition characterized by episodes of temporary immobility during transitions between wakefulness and sleep. This report describes a 30-year-old female presenting frequently with nightmares, sleep paralysis, and associated stress, successfully managed with a holistic approach incorporating yoga, meditation, chanting, and vitamin D3 supplementation. The patient's significant history of osteoporosis (in February 2019, bone density T-score <-2.5; vitamin D3: 17 nmol/L), drug-resistant tuberculosis, depression, and workplace stress compounded her symptoms. After managing her with a holistic management plan, she achieved sustained remission of sleep paralysis and improved overall health with increased bone density (bone density T-score in February 2021: -2.1; vitamin D3 in February 2020: 87 nmol/L), emphasizing the importance of addressing psychological, lifestyle, and nutritional factors in such cases.

## Introduction

Sleep paralysis (SP) is a condition characterized by the return of consciousness while the muscle atonia associated with rapid eye movement (REM) sleep persists [1]. Recurrent isolated sleep paralysis (RISP) is a harmless parasomnia characterized by repeated episodes of isolated sleep paralysis (at least two within six months) that cause significant distress, such as anxiety or fear linked to sleep or the bedroom environment [2]. This state can lead to significant fear and distress as individuals find themselves fully aware but unable to move any part of their body [3]. It is frequently accompanied by hallucinations, which are typically classified into two types: intruder and incubus hallucinations [4]. Intruder hallucinations involve a sensation of a threatening presence or person in close proximity [4]. In contrast, incubus hallucinations are marked by feelings of chest pressure, often accompanied by aggressive or sexual imagery [5]. These experiences are commonly associated with intense anxiety, immobility, and sensations of suffocation [5]. The general population has reported a 7.6% prevalence of sleep paralysis once in their lifetime, as compared to 28.3% in students and 31.9% in psychiatric patients [1]. Currently, there is no specific treatment available to directly address sleep paralysis during an active episode [5]. While efforts have been made to manage the underlying psychological and physical factors that may trigger episodes, no intervention has been proven to abort them in real-time [5]. Some studies have suggested potential treatments, such as pimavanserin, a selective 5-HT receptor inverse agonist, which may help alleviate hallucinations associated with sleep paralysis episodes [5]. Additionally, techniques like focused meditation and muscle relaxation therapies are proposed as management strategies [5]. Combining these approaches might, in theory, provide a stronger effect in preventing or reducing the frequency of sleep paralysis episodes [5].

A 30-year-old female presented with recurrent sleep paralysis episodes, nightmares, and associated distress, alongside a history of osteoporosis, drug-resistant tuberculosis, depression, and workplace stress. Detailed evaluations revealed low vitamin D3 levels, severe stress, and mild depression, while sleep apnea was ruled out. She was treated with vitamin D3 supplementation, lifestyle modifications including yoga, meditation, chanting, and dietary adjustments. Over three years, her symptoms resolved, her bone density improved to osteopenia, and she continues to lead a normal life with consistent well-being practices.

## Case presentation

A 30-year-old female came for private consultation with the first author with complaints of a series of nightmares and inability to move the whole body while asleep. She reported experiencing frequent distressing dreams almost every night for five years. Her medical history revealed a diagnosis of osteoporosis five months prior to her consultation with remarkably low bone mass density (T-score <2.5). A detailed history was taken, and routine blood investigations (complete blood count, liver function test, renal function test, serum calcium, and vitamin D3 levels) were advised to calm her apprehension and she was asked to bring them on the subsequent follow-up. The patient was diagnosed with drug-sensitive tuberculosis (TB) in December 2004 and underwent treatment in 2005 with a standard regimen consisting of isoniazid, rifampicin, pyrazinamide, and ethambutol for six months, achieving a cure. However, after a year, she experienced a relapse and was diagnosed with drug-resistant TB. Unfortunately, treatment records for the medications used during this relapse were not available. By January 2009, culture results showed no growth, and she was declared cured. The patient was diagnosed with depression by a certified psychiatrist in March 2017 and was treated for the same. She stopped taking medication without consultation in May 2017 as she couldn't process her emotions and had difficulty crying. She also had frequent panic attacks and chose not to take treatment for them. The first two episodes of sleep paralysis occurred before her diagnosis. Additionally, the patient reported experiencing workplace-related stress for two to three years. She had no history of excessive daytime sleepiness, epilepsy, cataplexy, snoring, or apnea. The patient had no significant family history. She had no difficulty forming friendships or engaging in social conversations. She did not experience social anxiety and maintained good relationships with her family, friends, and colleagues. At her workplace, she was well-regarded and had positive interactions with her peers. However, the competitive nature of her job and high expectations from demanding superiors contributed to significant workplace stress. She had no history of substance use, including alcohol or tobacco, and reported no other addictions. She strived to successfully manage to balance her professional and personal life. 

Screening with various scales like the Epworth Sleepiness Scale with a score of 6/19 indicating low daytime sleepiness; STOP-BANG and Berlin Scales indicating low risk for obstructive sleep apnea; Hamilton Depression Rating Scale (HDRS) with a score of 10 indicating mild depression; and Workplace Stress Scale with a score of 27 indicating severe stress [6-10]. On further exploration of the sleep paralysis episodes, the patient described three prominent events, each with a similar pattern of auditory hallucinations, paralysis, and intense fear. With each episode, the patient experiences increased intensity of fear and intruder hallucinations.

The first episode occurred in December 2016, during a business trip to a coastal city where she stayed alone in a hotel. She reported hearing whispers from a group of women around her bed, which intensified as she attempted to move but was unable to do so. She felt the voices getting louder and the group's presence coming closer. After several attempts, she regained movement, and the sensations dissipated. She checked her room and found herself alone. This was followed by intense fear and difficulty sleeping again.

The second episode happened two months later, in February 2017, while she was resting in her office's break room. She heard the familiar sound of her absent colleague’s voice and bangles, which intensified as it approached. Realizing the impossibility of the situation, she became petrified and, as in the previous event, was unable to move her body or call for help. The sensations dissipated on awakening.

The third episode occurred in April 2017 at her home. While sleeping alone in her room, she vividly saw a small girl playing near her bed, who then lay down near her legs. Shortly after, she felt an overwhelming presence, a man lying next to her who suddenly began choking her. Overcome by terror and an oppressive chest pressure, she was paralyzed, unable to scream or move. Despite this immobilization, she engaged in a mental and physical struggle to break free from the sensation. After a prolonged and desperate fight, she managed to force herself awake, at which point the horrifying experience ceased. Shaken and overwhelmed by fear, she immediately called her mother to stay with her for the rest of the night.

In response to her stress, the patient sought help from a local yoga center, where she was advised to practice meditation, chant the Gayatri mantra, and engage in daily yogasana for one hour. Following these interventions, she reported significant relief, with no further episodes until 2019, when she experienced a similar, isolated event.

After experiencing the fourth episode in 2019, the patient sought consultation with the first author to address her ongoing sleep disturbances and associated distress. The patient experienced distressing dreams where she found herself in uncomfortable situations, though not always frightening. At times, her dreams involved illogical or irrational scenarios. These dreams disrupted her sleep, preventing her from achieving deep, restorative rest. However, she did not habitually wake frequently during the night under normal circumstances. She was evaluated and asked to continue with her yoga and meditation practices. On examination, it was found that her body mass index (BMI) was 21.3 kg/m², blood pressure was 100/60 mmHg, heart rate was 85 bpm, respiratory rate was 16 breaths per minute, and SpO₂ was 100%. Her routine blood investigations were normal, and vitamin D3 levels were 17 nmol/L (severely deficient) baseline in Feb 2019. She had a baseline bone density T-score of less than -2.5, suggesting osteoporosis. Routine blood tests were within normal limits except for a low vitamin D3 level. She was prescribed multivitamin tablets (once daily) and vitamin D3 (tab cholecalciferol 60,000 IU once a week for six weeks). Her diet was also reviewed, and an appropriate diet plan that fulfilled her nutritional requirements was made. She was also told to continue her regular yogasana and pranayama for meditation and chanting of the Gayatri mantra. She was advised to read motivational books and listen to soothing music to encourage positivity. She was also advised to take an opinion from a psychiatrist concerning her depression and sleep paralysis, which she dismissed. She was advised to repeat vitamin D3 levels after six months and come for a follow-up.

At the follow-up visit in December 2019, her adherence to medication, meditation, and chanting practices was reviewed, and she reported engaging in at least 30 minutes of these activities daily to calm her apprehension regarding the night terrors. She had not experienced any further episodes of sleep paralysis since her initial consultation. However, there was no significant change in the frequency of distressful dreams. To address her persistent vitamin D3 deficiency, despite oral supplementation, she was administered an injectable bolus dose of intramuscular injection of vitamin D3 bolus injection (6 lac IU) after persistently low levels. 

Along with additional counseling to reinforce lifestyle changes, she was advised to monitor her vitamin D3 levels every six months. No oral supplementation was given during the initial six months after the bolus injection. In February 2020, her vitamin D3 levels were reported to be within the normal range, and she had no episodes of sleep paralysis or night terrors. Her vitamin D3 levels were 87 nmol/L in February 2020; her bone density T-score improved to -2.1, indicating osteopenia, in February 2021. This was a remarkable improvement with a single dose of injectable vitamin D3 supplementation. On the Hamilton Depression Rating Scale, her score reduced to 7, signifying mild depression. On the Workplace Stress Scale, she scored 32, indicative of moderate stress.

Over the past three years, along with consistently normal vitamin D3 levels (45 nmol/L in 2022) and improved bone density, the patient has remained free of sleep paralysis episodes and is leading a normal life. The patient was advised to consume a combined tablet containing calcium with a low dose of vitamin D3 (elemental calcium 500 mg and vitamin D3 250 IU). Regular yogasana (shuddhikriya and five types of pranayama), meditation, and chanting Gayatri mantra for 45-60 minutes have become integral to her daily routine, contributing to her overall well-being and mental resilience. Various differential diagnoses were reviewed: recurrent isolated sleep paralysis (RISP), obstructive sleep apnea, which is unlikely given low-risk scores on STOP-BANG and Berlin scales and absence of snoring or apnea, and post-traumatic stress disorder (PTSD), as she had auditory hallucinations and stress-related symptoms raised this possibility but did not meet diagnostic criteria. 

## Discussion

This case report highlights a unique presentation of RISP in a patient with a complex medical and psychological history. Such findings are common in RISP; some patients may even experience bedtime anxiety and bedroom avoidance behavior [11]. SP can be a distressing phenomenon, particularly when associated with hallucinations, as seen in this case. The patient's experience of auditory hallucinations and a perceived "presence" during episodes is consistent with the intruder hallucination subtype of sleep paralysis. In contrast, during the third episode, the incubus hallucination experienced by her contributed to her anxiety and fear of sleeping alone.

The differential diagnosis of RISP requires careful consideration of the patient's presentation, overall health, and medical history, with the need to rule out conditions such as focal epileptic seizures, atonic seizures, cataplexy, familial periodic paralysis (e.g., Andersen-Tawil syndrome), and transient compression neuropathies [1]. A major distinction lies between RISP and narcolepsy, as both conditions involve sleep paralysis, but narcolepsy also includes cataplexy and excessive daytime sleepiness, which are not present in most RISP cases [1]. If uncertainty arises, polysomnography and multiple sleep latency testing can help differentiate between the two [1].

Distinguishing RISP from psychiatric conditions can be difficult, as psychotic experiences are common, but RISP hallucinations are unique in their timing, occurring during sleep-wake transitions, and narrative structure [1]. Key differentiations include exploding head syndrome (EHS), where hallucinations are brief, loud, and undifferentiated, with no paralysis; nightmare disorder (ND), where there is no awareness or atonia, and dream imagery is always present; sleep terrors (STs), which are non-REM parasomnias without awareness of surroundings and involve screams, unlike RISP; nocturnal panic attacks (NPAs), which lack paralysis or dream imagery and have a sudden onset, in contrast to the fear, experienced in RISP after paralysis and hallucinations; post-traumatic stress disorder (PTSD), where flashbacks extend beyond sleep-wake transitions and paralysis is typically subjective; and psychotic disorders, where hallucinations are more pervasive and not confined to sleep-wake transitions, with intact reality testing during RISP episodes. These distinctions are crucial for accurate diagnosis and management [1].

Several factors in the patient’s medical and psychological history contributing to the development of RISP

Psychological Stress

Workplace stress and untreated panic attacks likely heightened her vulnerability to RISP episodes. Studies have shown that stress can disrupt REM sleep and trigger sleep paralysis [3].

Vitamin D Deficiency

While not directly linked to sleep paralysis, severe deficiency in vitamin D may contribute to fatigue, poor sleep quality, and mood disturbances, compounding the patient’s symptoms [12]. A systematic review statistically significantly showed that vitamin D supplementation improves sleep quality, while its effect on sleep disturbances and disorders wasn’t unanimous [13]. Vitamin D3’s role in sleep paralysis is an area that warrants further exploration due to its established connections with sleep regulation, neuromuscular function, and mental health.

Depression and Anxiety

The history of depression and panic attacks, coupled with moderate-to-severe depressive symptoms during initial consultation, aligns with existing literature suggesting a higher prevalence of RISP in individuals with mood disorders [3]. A case was reported with SP episodes while on sertraline, which was resolved by tapering the medicines [14]. In the case of our patient, her reluctance to see a psychiatrist and the lack of a record of medicines taken interfered with exploring this possibility.

Sleep Hygiene and Posture

Although not explicitly detailed, poor sleep hygiene and sleeping in a supine position are common triggers for sleep paralysis [1].

Multimodal approach

The multimodal approach adopted in this case demonstrates the importance of addressing both physical and psychological health in managing RISP: 

Lifestyle Modification

Regular practice of yogasana, pranayama, and meditation was central to the patient's recovery. These interventions likely improved her stress management, sleep quality, and overall mental health. Meditation, in particular, has been shown to stabilize REM sleep patterns and reduce the frequency of sleep paralysis episodes [15,16]. 

Vitamin D Supplementation

Correction of severe vitamin D deficiency with both oral and injectable forms not only addressed her bone health but also contributed to improved mood and energy levels, indirectly supporting her recovery. 

Counseling and Relaxation

Encouraging positive activities such as reading and listening to soothing music likely provided additional psychological benefits. 

The patient's complete resolution of sleep paralysis episodes over three years underscores the effectiveness of a holistic and patient-centered approach. This case further supports the role of stress management, meditation, and yoga in managing RISP. Her improvement in bone density and sustained normal vitamin D3 levels demonstrate the benefits of addressing underlying physical health issues alongside psychological ones. 

Challenges in the management of RISP

Patient Reluctance to Psychiatric Consultation

The patient's decision to forego psychiatric evaluation may have limited a deeper exploration of underlying mood disorders or post-traumatic stress. 

Lack of Sleep Studies

While the clinical diagnosis of RISP was evident, objective documentation through polysomnography could have further enriched the case. A limitation of this case is that sleep paralysis is unpredictable. In this instance, the patient experienced episodes without any witnesses. As a result, the diagnosis is based solely on the patient’s clinical history and self-reported experiences. Due to the unpredictable nature of sleep paralysis in this case, conducting a sleep study was not feasible. No sleep study or objective testing was conducted to support these reports, which limits the ability to observe and confirm the episodes directly.

Clinicians should consider RISP in patients presenting with distressing sleep-related events and emphasize non-pharmacological interventions like stress reduction and lifestyle modifications as primary management strategies. Additionally, addressing comorbid conditions like depression and vitamin deficiencies can significantly enhance outcomes, as demonstrated in this case. 

## Conclusions

In conclusion, this case emphasizes the need for an integrative approach to managing RISP, highlighting the role of lifestyle interventions, psychological support, and addressing underlying medical conditions. It serves as a reminder of the profound impact that simple, non-invasive measures can have on improving the quality of life in patients with complex clinical presentations.
